# Ectopic Expression of the Chinese Cabbage Malate Dehydrogenase Gene Promotes Growth and Aluminum Resistance in *Arabidopsis*

**DOI:** 10.3389/fpls.2016.01180

**Published:** 2016-08-03

**Authors:** Qing-Fei Li, Jing Zhao, Jing Zhang, Zi-Hui Dai, Lu-Gang Zhang

**Affiliations:** ^1^State Key Laboratory of Crop Stress Biology for Arid Areas, College of Horticulture, Northwest A&F UniversityYangling, China; ^2^College of Horticulture, Shanxi Agricultural UniversityTaigu, China

**Keywords:** malate dehydrogenase, aluminum stress, growth potential, Chinese cabbage, overexpression, *Arabidopsis*

## Abstract

Malate dehydrogenases (MDHs) are key metabolic enzymes that play important roles in plant growth and development. In the present study, we isolated the full-length and coding sequences of *BraMDH* from Chinese cabbage [*Brassica campestris* L. ssp. *pekinensis* (Lour) Olsson]. We conducted bioinformatics analysis and a subcellular localization assay, which revealed that the *BraMDH* gene sequence contained no introns and that BraMDH is localized to the chloroplast. In addition, the expression pattern of *BraMDH* in Chinese cabbage was investigated, which revealed that *BraMDH* was heavily expressed in inflorescence apical meristems, as well as the effect of *BraMDH* overexpression in two homozygous transgenic *Arabidopsis* lines, which resulted in early bolting and taller inflorescence stems. Furthermore, the fresh and dry weights of aerial tissue from the transgenic *Arabidopsis* plants were significantly higher than those from the corresponding wild-type plants, as were plant height, the number of rosette leaves, and the number of siliques produced, and the transgenic plants also exhibited stronger aluminum resistance when treated with AlCl_3_. Therefore, our results suggest that *BraMDH* has a dramatic effect on plant growth and that the gene is involved in both plant growth and aluminum resistance.

## Introduction

Malate plays a key role in plant nutrition and metabolism, as an important intermediate product (Schulze et al., 2002; Scheibe, 2004) and is generated by malate dehydrogenases (MDHs: EC 1.1.1.82 or EC 1.1.1.37), which function as oxidoreductases that catalyze the reversible reactions of malate and oxaloacetic acid, using NAD^+^ or NADP^+^ as coenzymes. The occurrence of MDHs is widespread in plants, animals, and microorganisms. In plants, several MDH isoforms possess specific subcellular locations and coenzymes. According to their subcellular location, plant MDHs are classified as mitochondrial, peroxisomal, plastidial, and cytosolic MDHs (Gietl, 1992). In recent years, different MDH isoforms have been reported in different species (Miller et al., 1998; Trípodi and Podestá, 2003; Yao et al., 2011; Abd El-Moneim et al., 2015; Zhang Bao et al., 2015). In the model plant *Arabidopsis thaliana*, nine MDHs have been identified, including two peroxisomal MDHs (Pracharoenwattana et al., 2007; Eubel et al., 2008), two mitochondrial MDHs (Millar et al., 2001; Tomaz et al., 2010), and two plastidial MDHs (Berkemeyer et al., 1998; Hebbelmann et al., 2012), as well as three cytosolic MDHs (CMDH1, 2, and 3) that were predicted in The *Arabidopsis* Information Resource^1^.

The different MDH isoforms play a variety of roles that depend on their subcellular localization and the metabolic activities that occur in their respective cellular compartments. For example, mitochondrial MDHs are key enzymes in tricarboxylic acid cycles and have been reported to be involved in leaf respiration and photorespiration (Tomaz et al., 2010). Peroxisomal MDHs have been proposed to be involved in fatty acid β-oxidation, by supplying the electron acceptor NAD^+^ via the catalyzation of oxaloacetic acid to malate (Pracharoenwattana et al., 2007), and were also involved in photorespiration, by oxidating malate to generate NADH (Reumann and Weber, 2006). In addition, two MDH isoforms have been reported in plastids, including both NADP-dependent and NAD-dependent MDHs (pdNADP-MDH and pdNAD-MDH) (Berkemeyer et al., 1998; Hebbelmann et al., 2012). Interestingly, pdNADP-MDH is strictly a light-redox enzyme, which is only activated in the light (Berkemeyer et al., 1998; Scheibe, 2004), and in C_3_ plants, the pdNADP-MDH functions as an important “malate valve”, by regulating the reducing equivalents (malate) to balance the NADPH/ATP ratio (Scheibe, 1987), whereas in C_4_ plants, pdNADP-MDH is involved in daytime CO_2_ fixation, by catalyzing the primary CO_2_ fixation product oxaloacetic acid to the stable CO_2_ product malate (Scheibe, 1987). However, pdNAD-MDH is active under both light and dark conditions (Berkemeyer et al., 1998), and in *Arabidopsis*, pdNAD-MDH has been demonstrated to play critical roles in seed development and heterotrophic metabolism (Beeler et al., 2014; Selinski et al., 2014). For example, *Arabidopsis* mutants that lacking the *pdNAD-MDH* exhibit defective embryo development, and the disruption of *pdNAD-MDH* expression results in *Arabidopsis* plants with abnormal phenotypes, such as reduced chlorophyll and photosynthetic levels, damaged chloroplast ultrastructure, and dwarfed seedlings (Beeler et al., 2014; Selinski et al., 2014). Other research has reported that overexpression of a plastidial *MDH* gene promotes the growth of transgenic apple plants, especially of roots, which was linked to improved photosynthesis and reduced levels of abscisic acid (Wang et al., 2015). The overexpression of a cytosolic *MDH* gene in apple was reported to contribute to increased MDH activity and malate accumulation (Yao et al., 2011).

Several reports have indicated that MDHs have a close relationship with stress resistance (Cushman, 1993; Wang et al., 2010); however, direct evidence for this is scarce, and although several studies have reported that overexpression of the *Arabidopsis* plastidial and cytosolic MDH genes could improve the resistance of transgenic tomato to aluminum and acidic soil (Zhang et al., 2008; Wang et al., 2010), it remains unclear whether other MDH isoforms have the same effect. Furthermore, although the roles of *MDH* genes have been explored for many years, most of these studies have focused on model plants. However, studies of MDH function should also include non-model plants, such as Chinese cabbage [*Brassica campestris* L. ssp. *pekinensis* (Lour) Olsson], which is a popular vegetable in many regions of the world. In addition, further studies are also needed to elucidate the role of MDHs in aluminum tolerance. Because aluminum is the third most abundant element after oxygen and silicon, plant roots are almost always exposed to aluminum in some form, and although most aluminum occurs as oxides and aluminosilicates, which are harmless, the element becomes toxic to plants in acidic soils (Ma et al., 2001). For example, several studies have reported that Al^3+^ toxicity inhibits root growth and nutrient uptake (Delhaize and Ryan, 1995; Lopez-Bucio et al., 2000).

Therefore, in the present study, we analyzed the expression of the *BraMDH* in different tissues of Chinese cabbage. In addition, we also investigated the role of *BraMDH* in plant growth, development, and aluminum tolerance by overexpression *BraMDH* in *Arabidopsis*.

## Materials and Methods

### Plant Materials and Growing Conditions

Chinese cabbages (line 97C16-3) were planted and maintained in the greenhouse under natural light conditions (Yangling, Shaanxi).

*Arabidopsis thaliana* ecotype Columbia (Col-0) was used in the production of transgenic plants. Seeds were surface sterilized and sown on Murashige and Skoog (MS) medium (Murashige and Skoog, 1962) with 2% sucrose, 0.8% agar, and a pH of 5.7 (adjusted with NaOH). For uniform germination and to break dormancy, the seeds were initially incubated on MS medium for 2–3 days in the dark at 4°C, after which they were transferred to a growth chamber and incubated at 22°C and 50–70% relative humidity, with a photoperiod of 12 h light/12 h dark. The light intensity of the two plant culturing shelves in the growth chamber are slightly different; the light intensity of the first shelf, which held the WT1 and TL3 plants, was 98 μmol m*^-^*^2^s*^-^*^1^, whereas the light intensity of the second shelf, which held WT2 and TL9, was 115 μmol m*^-^*^2^s*^-^*^1^.

### Cloning and Bioinformatics Analysis of *BraMDH*

Genomic DNA was extracted from Chinese cabbage leaves using a modified CTAB protocol (Allen et al., 2006) and total RNA was extracted using a Trizol^TM^ kit (Invitrogen, Carlsbad, CA, USA), according to the manufacturer’s instructions. RNA purification and first-strand cDNA synthesis were conducted using the Prime Script^TM^ RT Reagent Kit (Takara, Dalian, China). The gene-specific primers *BraMDH*-F (5′-ATGGCAGCAGCTTCTTCGATTTC-3′) and *BraMDH*-R (5′- TTAGTTAGCAGGTTTGTTTGCGAATG-3′) were used to amplify the full-length and coding sequences of *BraMDH*. The PCR products were gel purified and cloned into the pMD18-T vector (Takara, Dalian, China). The positive clones were sequenced by Sangon Biotech Company Limited (Shanghai, China).

Once the sequences were obtained, the DNAMAN software was used to analyze the nucleotide and amino acid sequences of *BraMDH* and the online software Tmpred^2^ was used to predict the transmembrane domain.

### Testing Tissue-Specific Expression of *BraMDH*

To investigate the expression pattern of *BraMDH* in Chinese cabbage, the total RNA were extracted from leaves, flower buds, flowers, inflorescence stems, inflorescence apical meristems, and young siliques using a Trizol^TM^ kit (Invitrogen, Carlsbad, CA, USA), according to the manufacturer’s instructions. RNA purification and first-strand cDNA synthesis were conducted using a PrimeScript^TM^ RT Reagent Kit with gDNA Eraser for Real Time (Takara, Dalian, China), according to the manufacturer’s instructions. The gene specific primer *GAPDH*-F (5′-TAACTGCCTTGCTCCACTTGC-3′) and *GAPDH*-R (5′- CGGTGCTGCTGGGAATGAT-3′) were used to amplify the *GAPDH* (GO0048316), which was used as an internal control. The specific primer q*BraMDH-*F (5′-GGACCAGATTCCTCTTCTTTCGC-3′) and q*BraMDH*-F (5′- TGATGTTCAATCCGTAGGGCTTC-3′) were used to amplify the *BraMDH*. The real-time PCR reaction system was composed of 20 μL mixture that consisted of 10 μL SYBR Premix Ex Taq^TM^ II (Takara, Dalian, China), 0.8 μL forward primer (10 μM), 0.8 μL reverse primer (10 μM), 2 μL diluted cDNA (200 ng), and 6.4 μL ddH_2_O. All reactions were performed in triplicate. The real-time PCR was conducted in a Bio-Rad IQ5 instrument (Foster City, CA, USA), using a two-step thermal cycling program with the following conditions: 1 cycle of 95°C for 30 s, 40 cycles of 95°C for 5 s, and 62°C for 30 s. Serial dilutions of cDNA were used to calibrate a standard curve for each gene, in order to ensure that the amplification efficiency of actin and target gene primers were almost equal and nearly 100%. The relative expression levels of *BraMDH* were normalized with that of *GAPDH* using the 2^-ΔΔCt^ method (Livak and Schmittgen, 2001).

### Subcellular Localization of BraMDH

In order to determine the subcellular location of BraMDH, we used the online software iPSORT^3^ and TargetP^4^ to predict its transit peptide. In addition, we also constructed a BraMDH-green fluorescent protein (GFP) fused expression vector and transiently transformed into isolated *Arabidopsis* protoplasts. Firstly, The coding region of *BraMDH* was amplified using the gene specific primers GFP-*BraMDH*-F (5′-GCTCTAGAATGGCAGCAGCTTCTTCGATTTC-3′ with *XbaI* site under lined) and GFP-*BraMDH* -R (5′- AAGGTACCGTTAGCAGGTTTGTTTGCGAATG -3′ with *KpnI* site underlined). The amplified products were cloned into the PBI221-GFP vector. The constructed vector was then transformed into *Arabidopsis* protoplasts, which were isolated from callus as described previously (Yoo et al., 2007) with some modification. The transient expression of the BraMDH-GFP fusion protein was observed using a Nikon A1R confoeal laser scanning microscopy (Tokyo, Japan).

### Construction of the Overexpression Vector and Its Genetic Transformation into *Arabidopsis*

The coding region of *BraMDH* was amplified using the gene specific primers *BraMDH*-F (5′-GCTCTAGAATGGCAGCAGCTTCTTCGATTTC-3′ with *XbaI* site underlined) and *BraMDH*-R (5′- AACTGCAGTTAGTTAGCAGGTTTGTTTGCGAATG-3′ with *PstI* site underlined), and the amplified product was cloned into the pCAMBIA2301-35S-Nos (Kanr) vector, which was a gift from Professor Yuke He (Shanghai Institutes for Biological Science, Chinese Academy of Sciences). The recombinant vector, p2301-*BraMDH* was then transformed into *Agrobacterium tumefaciens* strain GV3101 and was confirmed by PCR, restriction enzyme digestion, and sequence analysis. Subsequently, the p2301-*BraMDH* vector was transformed into the *Arabidopsis* ecotype Columbia (Col-0) using the floral dip method (Clough and Bent, 1998). Seeds of the transgenic plants were screened on MS medium (2% sucrose, 0.8% agar) containing 50 μg⋅mL^-1^ kanamycin, and the T_1_ positive plants were transferred to a substrate mixture of peat, vermiculite, and pearlite (3:1:0.5, v/v). The surviving transformants were further confirmed by amplifying the target gene *BraMDH*. The T_2_ seeds from positive T_1_ transformants were individually harvested and sown on MS medium (2% sucrose, 0.8% agar) that contained 50 μg⋅mL^-1^ kanamycin, in order to screen for T_2_ transgenic lines that corresponded to a 3:1 segregation ratio of survival: lethal. T_3_ seeds were harvested from the T_2_ transgenic lines that exhibited 3:1 segregation ratios, and the T_3_ lines that exhibited 100% kanamycin resistance were considered as homozygous.

### Analysis of *BraMDH* Expression in Transgenic *Arabidopsis*

Total RNA was isolated from the rosette leaves of wild-type (WT) and transgenic *Arabidopsis* lines (T3 and T9) using a Trizol^TM^ kit according to the manufacturer’s instruction (Invitrogen, Carlsbad, CA, USA), and a PrimeScript^TM^ RT Reagent Kit with gDNA Eraser for Real Time (Takara, Dalian, China) was used for cDNA synthesis. Real-time PCR was performed using SYBR^®^ Premix Ex Taq^TM^ II fluorochrome (Takara, Dalian, China). The specific primers *Tub-*F (5′-TTTGTGCTCATCTTGCCACGGAAC-3′) and *Tub-*R (5′-CTCAAGAGGTTCTCAGCAGTACC-3′) were used to amplify *Tub* (AT5G62690), which was used as an internal reference gene. The specific primers q*BraMDH*-F and q*BraMDH*-R were used to amplify *BraMDH*. The amplification efficiencies of the primers were tested as described above. The real-time PCR was conducted using the following conditions: 1 cycle of 95°C for 30 s, 40 cycles of 95°C for 5 s and 62°C for 30 s; and all reactions were performed in triplicate. The relative expression of *BraMDH* were calculated using the 2^-ΔΔCT^ method (Livak and Schmittgen, 2001).

### Growth Phenotypes of Transgenic *Arabidopsis* Plants

Seeds of the homozygous transgenic *Arabidopsis* lines (TL3 and TL9) and their corresponding wild-type lines (WT1 and WT2) were sown on MS medium (2% sucrose, 0.8% agar), and after 10–12 days, the seedlings were transferred to pots filled with the same weight of substrate mixture. The occurrence of bolting was recorded for each plant at 30 and 35 days after the seedlings were transplanted. In addition, the plant height, fresh and dry weights of aerial tissue, number of rosette leaves, and number of siliques were recorded at 45 days after the seedlings were transplanted.

### AlCl_3_ Treatment of *Arabidopsis* Plants

Both homozygous transgenic plants (TL3 and TL9) and wild-type plants were used for AlCl_3_ treatments. For the first treatment, sterilized seeds were sown on MS medium (2% sucrose, 0.8% agar) and grown for 10 days in the growth chamber, after which the WT and homozygous transgenic seedlings (*n* = 15 each) were transferred to 0, 50, and 100 μM AlCl_3_ solutions. After 3 days of treatment, all seedlings cultured in 0, 50, and 100 μM AlCl_3_ solutions were investigated, and the root activity of WT and homozygous transgenic seedlings cultured in 50 μM AlCl_3_ were determined using triphenyl tetrazolium chloride (TTC) reduction method as described previously (Clemensson-Lindell, 1994; Mugai et al., 2000). TTC reduction method can examine the dehydrogenase activity, and thus it is often used to assess the root activity (Clemensson-Lindell, 1994; Mugai et al., 2000). Firstly, the roots of the pre- and post-treatment WT and homozygous transgenic seedlings were cut and put into centrifugal tubes with 1 mL 1% (m/v) TTC and 1 mL 0.1 mol/L Na_2_HPO_4_-NaH_2_PO_4_ solution, and after that the tubes were kept at 37°C for 2 h. Then, the roots were observed by a dissecting microscope (Olympus, Tokyo, Japan). For the second treatment, both homozygous transgenic lines (TL3 and TL9) and their corresponding WT plants were cultivated in the pots filled with the same weight of substrate mixture, and watered with equal volumes of water. Then, after reaching the rosette stage, the plants were subjected to a water-deficit condition, after which they were irrigated with a 150 μM AlCl_3_ solution (Wang et al., 2010; Ding et al., 2013), and the SPAD values and chlorophyll fluorescence of all the plants were measured both before treatment (pre-treatment) and 2 days (post-treatment) after treatment. In addition, the morphological changes in the *Arabidopsis* plants were observed continuously during the 5-day treatment. The SPAD values and chlorophyll fluorescence parameters were measured using a SPAD-502 chlorophyll meter (Konica Minolta, Japan) and a portable PAM-2500 system (Walz Heinz, Effeltrich, Germany).

## Results

### *BraMDH* Nucleotide Sequence and Protein Properties

**FIGURE 1:**
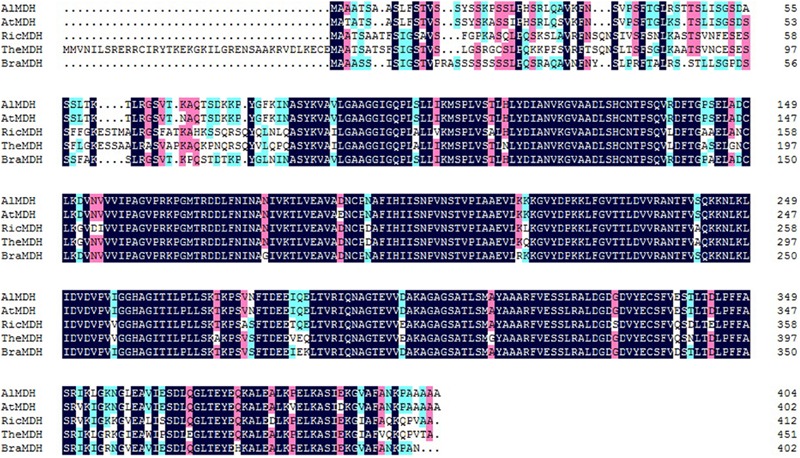
**Alignment of the predicted BraMDH protein sequence and other known MDH sequences.** The GenBank accession numbers of these proteins are as follows: AlMDH (XP_002875844.1), AtMDH (AT3G47520), RicMDH (XP_002514750.1), TheMDH (XP_007037368.1); Al, *Arabidopsis lyrata*; At, *Arabidopsis thaliana*; Ric, *Ricinus communis*; The, *Theobroma cacao*; Bra, *Brassica campestris*.

BLAST analysis of the full-length and coding sequences of *BraMDH* indicated that the gene contained no introns (Supplementary Figure S1). Bioinformatics analysis indicated that *BraMDH* encodes a 402-amino acids transmembrane protein that has a single transmembrane domain, molecular mass of 42.3 kDa, and isoelectric point (pI) of 8.347. In addition, multiple sequences alignment analysis revealed that the BraMDH amino acid sequence shared 89, 88, 78, and 78% identity with AlMDH (XP_002875844.1), AtMDH (AT3G47520), RicMDH (XP_002514750.1), and TheMDH (XP_007037368.1), respectively (**Figure 1**). The BraMDH amino acid sequence shared high homology with the *A. lyrata* and *A. thaliana* chloroplast MDH protein (AlMDH and AtMDH), which provides justification for the subcellular localization of BraMDH in *Arabidopsis* protoplasts.

### Localization of BraMDH in Chloroplast

**FIGURE 2:**
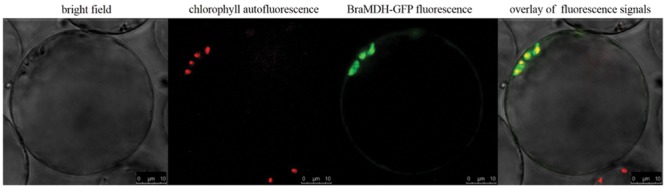
**Subcellular localization of BraMDH in *Arabidopsis* protoplasts.** From left to right: bright field, chlorophyll autofluorescence, BraMDH-GFP fused fluorescence, and the overlay of fluorescence signals. Bars = 10 μm.

Both iPSORT and TargetP identified a putative chloroplast transit peptide in the BraMDH amino acid sequence, and the co-localization of chlorophyll autofluorescence and BraMDH-GFP fusion protein fluorescence in *Arabidopsis* protoplasts confirmed that BraMDH was a chloroplast protein (**Figure 2**).

### The Expression of *BraMDH* in Chinese Cabbage

**FIGURE 3:**
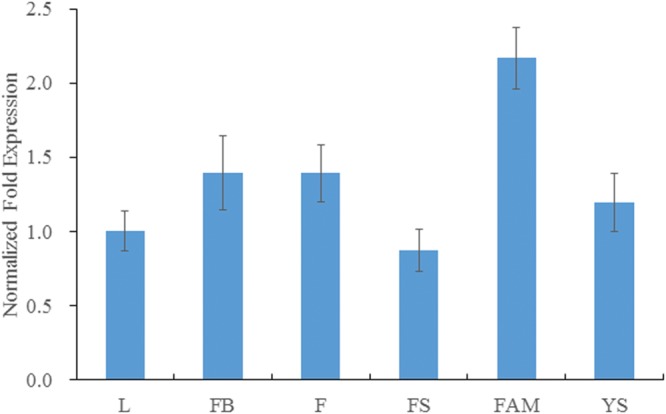
**Expression of *BraMDH* in Chinese cabbage.** L, leaves; FB, flower buds; F, flowers; FS, inflorescence stems; FAM, inflorescence apical meristems; YS, young siliques. Values indicate mean ± SD (*n* = 3).

The expression level of *BraMDH* was highest in the inflorescence apical meristem, followed by the levels observed in flower buds, flower, young siliques, leaves, and inflorescence stems, respectively (**Figure 3**), and the expression level in inflorescence apical meristem was more than twice that in leaves. The expression level of *BraMDH* in flower buds and flowers were almost identical, and the expression level in young siliques was higher than that in leaves. It has been reported that the *pdNAD-MDH* gene in *Arabidopsis* is crucial for seed development (Beeler et al., 2014; Selinski et al., 2014). Indeed, our results indicate that the *BraMDH* gene may play an important role in the reproductive organs of Chinese cabbage as well, especially during flower development and growth.

### *BraMDH* Expression in Transgenic *Arabidopsis* Plants

**FIGURE 4:**
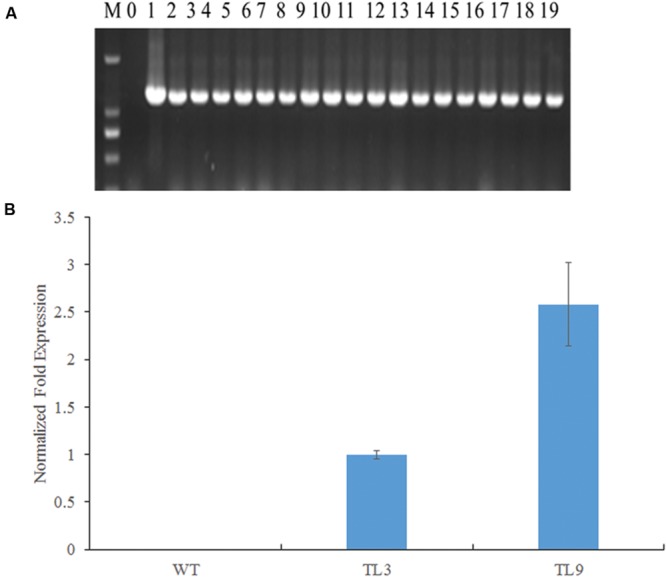
**Identification of *BraMDH*-expressing transgenic *Arabidopsis* lines. (A)** Electrophoresis gels of PCR products amplified from independent transgenic *Arabidopsis* lines (M: DL2000, 0: wild-type, 1: plasmid positive control, 2–19: transgenic plants). **(B)** Normalized fold expression of *BraMDH* in rosette leaves of wild-type (WT) and homozygous transgenic lines (TL3 and TL9). Values indicate mean ± SD (*n* = 3).

The full-length *BraMDH* fragment was amplified from all surviving T_1_ transformants (**Figure 4A**), whereas no gene fragments were detected in the WT plants, which together indicated that the *BraMDH* gene was successfully transmitted into the *Arabidopsis* genome. Then, after screening T_2_ lines which corresponded to a 3:1 segregation ratio of survival: lethal, we eventually identified two T_3_ homozygous transgenic lines (TL3 and TL9), as indicated by their 100% kanamycin resistance. In addition, we also confirmed that *BraMDH* was expressed in the TL3 and TL9 lines and found that the expression of *BraMDH* was higher in the TL9 line than in the TL3 line (**Figure 4B**). Thus, the transgenic lines TL3 and TL9 were considered homozygous and were used in subsequent studies.

### Overexpression of *BraMDH* Induces Early Bolting in Transgenic *Arabidopsis* Plants

**FIGURE 5:**
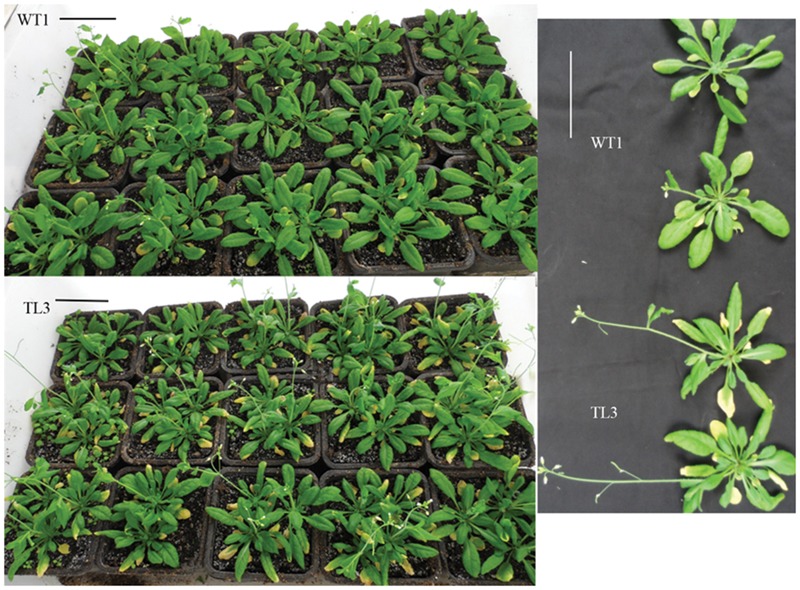
**Bolting in wild-type (WT1) and homozygous transgenic lines (TL3) plants.** Bolting of WT1 and TL3 plants after 35 days of growth in a substrate mixture of peat, vermiculite, and pearlite (3:1:0.5, v/v). Bars = 5 cm. The bolting of WT2 and TL9 plants is shown in Supplementary Figure S2.

After 30 days of growth, we found that none of the WT1 or WT2 plants had bolted, whereas 66 and 59% of the TL3 and TL9 plants had bolted, respectively; and after 35 days of growth, only to 57 and 60% of the WT1 and WT2 plants had bolted, respectively, whereas all of the transgenic plants had bolted. In addition, we also observed that the inflorescence stems of the WT1, WT2, TL3, and TL9 lines were 4.2 ± 3.0 cm, 7.6 ± 2.4 cm, 10.1 ± 3.7 cm, and 13.5 ± 1.2 cm, respectively, which clearly indicated that the inflorescence stems of the transgenic lines were significantly taller than those of the corresponding WT plants (**Figure 5**; Supplementary Figure S2). These results indicate that the overexpression of *BraMDH* contributes to early bolting *Arabidopsis*.

### Overexpression of *BraMDH* Enhanced Growth of Transgenic *Arabidopsis* Plants

At 45 days after the experimental seedlings were transferred to the substrate mixture, we found that plant height, rosette leaf number, siliques number, and aerial tissue weights of both transgenic lines were greater than those of their corresponding WT controls (**Table 1**). In particular, the fresh weights of the aerial tissue from TL3 and TL9 plants were 22 and 19% greater than those of their corresponding WT plants, respectively, and the difference both reached significant level (*p* < 0.05). In addition, the dry weights of the aerial tissue from TL3 and TL9 were 29 and 25% greater than those of their corresponding WT plants, respectively, and the difference both reached extremely significant level (*p* < 0.01) (**Table 1**). These data indicate that the overexpression of *BraMDH* enhanced the vegetative growth of the transgenic *Arabidopsis* plants, which likely contributed to the earlier bolting phenotype via enhanced nutritional resources. It is also interesting to note that no difference was observed in the dry weight/fresh weight ratio of aerial tissue from the WT and transgenic plants, which suggested that the overexpression of *BraMDH* did not affected the balance between dry material accumulation and water absorption.

**Table 1:** Growth traits of wild-type (WT1 and WT2) and homozygous transgenic (TL3 and TL9) *Arabidopsis* plants.

Line	Plant height (cm)	Rosette leaf number	Silique number	Aerial tissue fresh weight (g)	Aerial tissue dry weight (g)	Aerial tissue dry weight /fresh weight
WT1	36.17 ± 2.90	13.8 ± 1.1	72.1 ± 11.2	0.97 ± 0.16	0.14 ± 0.25	0.15 ± 0.01
TL3	39.03 ± 2.08^∗^	16.1 ± 1.2^∗∗^	83.3 ± 11.6^∗^	1.18 ± 0.21^∗^	0.18 ± 0.29^∗∗^	0.15 ± 0.01
WT2	36.29 ± 1.05	15.3 ± 1.3	85.1 ± 13.1	0.81 ± 0.11	0.12 ± 0.01	0.16 ± 0.01
TL9	36.42 ± 2.31	15.5 ± 0.7	87.4 ± 10.2	0.96 ± 0.10^∗^	0.15 ± 0.02^∗∗^	0.16 ± 0.02

*^∗^Significant difference (*P* < 0.05), ^∗∗^Highly significant difference (*P* < 0.01).*

### Overexpression of *BraMDH* Enhanced Al Tolerance of *Arabidopsis*

**FIGURE 6:**
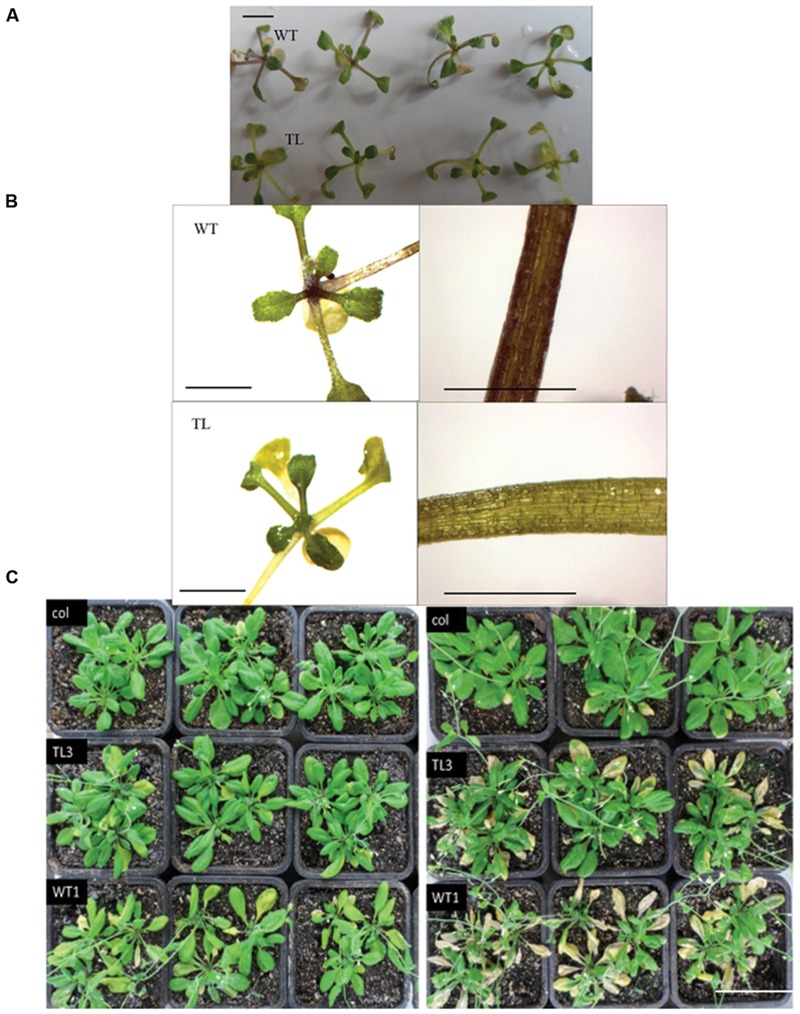
**Effect of aluminum stress on the growth of wild-type (WT1) and homozygous transgenic line (TL3) plants. (A,B)** Whole plants and leaf stalk of seedlings treated with 50 μM AlCl_3_ (Treatment 1), bars = 3 mm. **(C)** Rosette-stage WT1 and TL3 plants treated with 150 μM AlCl_3_ (Treatment 2) for 2 days (left) and 5 days (right), bars = 5 cm. Col: untreated wild type plants, WT: treated wild-type plants, TL: treated transgenic plants. The results of WT2 and TL9 plants are shown in Supplementary Figure S3.

After 3-day AlCl_3_ exposure of the first treatment, we found that all the seedlings cultured in 0 μM AlCl_3_ (distilled water) grew normally, that all the seedlings cultured in 100 μM AlCl_3_ solution were nearly dead, and that the WT and transgenic seedlings cultured in 50 μM AlCl_3_ solution exhibited significantly different phenotypes (**Figures 6A,B**). More specifically, we found that 63% of the WT seedlings had purple petioles, whereas only 40% of the transgenic lines had purple petioles. In addition, no significant difference of the root activity, which was assessed by the dehydrogenase activity according to the reduction level of TTC, had been found between the pre-treatment WT and transgenic seedlings; whereas the red color of transgenic seedlings was darker than that of WT (Supplementary Figure S4), and which indicated that the root of transgenic lines have greater TTC reduction level and dehydrogenase activity, after they had been exposed in 50 μM AlCl_3_ solution for 3 days (Steponkus, 1971).

Meanwhile, in the second treatment, we found that the rosette leaves of WT1 and WT2 plants became wilted after 2 days of treatment and then turned yellow and dry after 5 days. However, the wilting of the transgenic plants was less pronounced, and after 5 days of treatment, the color of the TL3 and TL9 leaves remained green and did not appear to dry out (**Figure 6C**; Supplementary Figure S3). In order to define the reason for wilting and chlorosis phenotypes, the chlorophyll content (SPAD value) and chlorophyll fluorescence of WT1, WT2, TL3, and TL9 treated with 150 μM AlCl_3_ solution were measured (**Table 2**). The mean SPAD values of WT1, WT2, TL3, and TL9 leaves were 25.7, 28.8, 28.0, and 30.4, respectively, before treatment. Obviously, the SPAD values of transgenic lines were higher than those of the corresponding WT lines. However, after being exposed to AlCl_3_ for 2 days, the SPAD values of WT1, WT2, TL3, and TL9 lines dropped to 16.3, 16.8, 23.1, and 23.6, respectively. The decreased ratio of SPAD values of WT1 and WT2 were 36.6 and 41.7%, respectively, which were higher than 17.5 and 22.4% in TL3 and TL9. These indicated that, under AlCl_3_ treatment, the chlorophyll content of transgenic plant leaves was more stable than that of WT leaves. Furthermore, in regards to chlorophyll fluorescence, we observed no significant differences, in pre- and post-treatment measurements of non-photochemical quenching (NPQ) and variable fluorescence/maximum fluorescence yield (*F*_v_/*F*_m_) of either the WT or transgenic plants (**Table 2**). However, the pre-treatment values of ground state fluorescence (*F*_o_), maximum fluorescence (*F*_m_), and photochemical quenching (*q*P) were slightly higher in transgenic plants than that in WT plants; and the *F*_o_, *F*_m_, and *q*P values of WT plants decreased significantly in response to the AlCl_3_ treatment, along with the chlorophyll content, whereas the parameters of the transgenic plants did not change significantly (**Table 2**). As a result, the post-treatment *F*_o_, *F*_m_, and *q*P values of TL3 and TL9 plants were significantly higher than those of WT1 and WT2 (**Table 2**), which indicated that the greater aluminum resistance of transgenic lines was associated with the higher light absorption, but not photosynthetic efficiency.

**Table 2:** Effect of aluminum (AlCl_3_) stress on the chlorophyll fluorescence parameters of wild-type (WT1 and WT2) and homozygous transgenic (TL3 and TL9) *Arabidopsis* plants.

Line	Pre-treatment (0 days)					Post-treatment (2 days)				
	*F*_o_	*F*_m_	*F*_v_/*F*_m_	*q*P	NPQ	*F*_o_	*F*_m_	*F*_v_/*F*_m_	*q*P	NPQ
WT1	0.79 ± 0.03	3.75 ± 0.14	0.79 ± 0.00	0.82 ± 0.03	0.22 ± 0.07	0.76 ± 0.03	3.45 ± 0.08	0.78 ± 0.00	0.76 ± 0.04	0.21 ± 0.03
TL3	0.82 ± 0.05	3.90 ± 0.21	0.79 ± 0.00	0.84 ± 0.03	0.22 ± 0.06	0.85 ± 0.08^∗^	3.93 ± 0.38^∗^	0.78 ± 0.00	0.83 ± 0.02^∗∗^	0.19 ± 0.03
WT2	0.79 ± 0.10	3.74 ± 0.32	0.79 ± 0.02	0.76 ± 0.04	0.23 ± 0.03	0.75 ± 0.05	3.28 ± 0.39	0.77 ± 0.02	0.65 ± 0.09	0.20 ± 0.02
TL9	0.80 ± 0.04	3.93 ± 0.18	0.80 ± 0.01	0.77 ± 0.05	0.22 ± 0.03	0.82 ± 0.09	3.76 ± 0.16^∗^	0.78 ± 0.02	0.75 ± 0.03^∗^	0.22 ± 0.01

*^∗^Significant difference (*P* < 0.05), ^∗∗^Highly significant difference (*P* < 0.01).*

*F_*o*_, ground state fluorescence; *F*_*m*_, maximum fluorescence; *F*_*v*_, variable fluorescence; *q*P, photochemical quenching; NPQ, non-photochemical quenching.*

## Discussion

Malate is an important intermediate product, and plays a key role in plant nutrition, metabolism, and cellular energy supply (Schulze et al., 2002). Because MDHs are crucial for generating malate, they have drawn increasing attentions from the scientific community (Schulze et al., 2002; Scheibe, 2004), and many researchers have studied the function of MDH genes; however, most of these studies have been in the model plants (Gietl, 1992; Pracharoenwattana et al., 2007; Tomaz et al., 2010; Selinski et al., 2014), and the role of *MDH* genes in economic crops remains unclear. In the present study, we isolated *BraMDH* which located in chloroplast from Chinese cabbage and discovered that ectopic expression of *BraMDH* augmented the growth and aluminum resistance of transgenic *Arabidopsis* plants. More specifically, the transgenic *Arabidopsis* lines exhibited greater fresh and dry weights of aerial tissue, plant height, number of rosette leaves, and number of siliques than the WT plants, and in addition, the transgenic plants also bolted earlier and exhibited more stable chlorophyll content and fluorescence (*F*_o_, *F*_m_, and *q*P).

Previous studies have reported that malate valves act as a powerful system for balancing ATP/NAD(P)H ratio required in energy-consuming reactions within plant cell (Scheibe, 2004). Therefore, the changes observed in the transgenic plants could indicated that the overexpression of *BraMDH* contributes to the production of malate, which can be shuttled from organelles and be used as a base material in ATP production within chloroplast and mitochondria (Scheibe, 2004; Tomaz et al., 2010; Selinski et al., 2014) and, subsequently, accelerate plant growth and development. Rosette leaves are the main photosynthetic organ of *Arabidopsis* plants, therefore, enhanced plant growth (i.e., more rosette leaves) results in more photosynthetic products, which increased the yield of biomass, so that the transgenic plants exhibit augmented plant height, siliques number, and bolting phenomenon. Indeed, the transgenic *Arabidopsis* plants in present study exhibited increased chlorophyll content and fluorescence, as well as increased growth, which supports earlier finding that *Arabidopsis* plants lacking the plastid *NAD-MDH* gene showed reduced levels of chlorophyll and photosynthesis, damaged chloroplast ultrastructure, and the inability to develop seeds (Beeler et al., 2014; Selinski et al., 2014) and also indicates that the function of *BraMDH* is similar to that of *pdNAD-MDH* in *Arabidopsis*.

Interestingly, our study also demonstrated that *BraMDH* contributes to stress tolerance, which supports previous findings regarding plastidial and cytosolic *MDH* genes in transgenic tomato plants (Zhang et al., 2008; Wang et al., 2010). In the present study, we found that the overexpression of *BraMDH* enhanced the aluminum tolerance of transgenic *Arabidopsis* (**Figure 6**; Supplementary Figure S3) and that also increased the stability of the plants’ chlorophyll content and fluorescence over that of WT plants (**Table 2**). It follows, then, that the stability of chlorophyll content in transgenic plants could be correlated with aluminum resistance. Indeed, *F*_o_, which is the minimal fluorescence in PSII reaction centers, has a close relationship to the chlorophyll content (van Kooten and Snel, 1990), and both *F*_m_ and *q*P, which represent electron transfer and photosynthetic activity in PSII reaction centers (van Kooten and Snel, 1990), were reduced slightly than in treated WT plants. This indicates that the electron transfer and photosynthetic activity were reduced in PSII reaction centers of WT plants, but those of transgenic plants were not impacted. We further speculate that the wilting of leaves in the treated WT plants reduced the plants’ light absorption abilities, which then contributed to reduced *F*_o_ and *F*_m_ levels, whereas the more stable chlorophyll content, *F*_o_, and *F*_m_ of the transgenic lines allowed the transgenic plants to outperform the WT plants when under the AlCl_3_ stress. Overall, the reason for increase of the aluminum resistance in the transgenic *Arabidopsis* is speculated that overexpression of *BraMDH* could have catalyzed conversions of the oxaloacetic acid to malate and other organic acids. This statement could be supported indirectly by the greater dehydrogenases activity that reflected by the higher TTC reduction level of transgenic *Arabidopsis* under the AlCl_3_ stress (Supplementary Figure S4). It is well established that, when faced with aluminum stress, some plants are capable of releasing organic acids (e.g., citric acid, malate, and oxalate) into the rhizosphere and that the organic acids form non-toxic complexes with Al^3+^ to preventing the toxic element from entering the roots (Pellet et al., 1996; Ma et al., 2001; Kochian et al., 2004), and moreover, overexpression of *neMDH* in transgenic alfalfa was reported to enhance both organic acid synthesis and aluminum tolerance (Tesfaye et al., 2001).

## Conclusion

The present study demonstrates that overexpression of *BraMDH* promotes growth and enhances aluminum resistance in transgenic *Arabidopsis* plants. The results suggest that *BraMDH* possesses potential value for genetic breeding of high-yield and aluminum-resistance Chinese cabbage cultivars.

## Author Contributions

QL and LZ conceived the experiments; QL and JZo performed the experiments; JZg participated in gene cloning and sequence analysis; ZD participated in seedling culture; LZ contributed materials, reagents, and analysis tools; QL and LZ wrote the paper.

## Conflict of Interest Statement

The authors declare that the research was conducted in the absence of any commercial or financial relationships that could be construed as a potential conflict of interest.

## Footnotes

1 http://www.arabidopsis.org/index.jsp

2 http://www.ch.embnet.org/software/TMPRED_form.html

3 http://ipsort.hgc.jp/

4 http://www.cbs.dtu.dk/services/TargetP/
