# Supplementary material for: Ectopic Expression of the Chinese Cabbage Malate Dehydrogenase Gene Promotes Growth and Aluminum Resistance in Arabidopsis
Source: Front Plant Sci. 2016 Aug 3;7:1180. doi: 10.3389/fpls.2016.01180 (PMC4971079; doi:10.3389/fpls.2016.01180)
Supplement: Supplementary file 1 [file Presentation_1.PDF]

Supplementary Material

Ectopic Expression of the Chinese Cabbage Malate Dehydrogenase Gene Promotes Growth and Aluminum Resistance in *Arabidopsis*

Qing-Fei Li[1], Jing Zhao[1], Jing Zhang[2], Zi-Hui Dai[1], and Lu-Gang Zhang[1*]

*** Correspondence:** Lu-Gang Zhang: lugangzh@163.com

```
ATGGCAGCAGCTTCTTCGATTTCAATTGGATCAACCGTTCCCAGAGCCTCCTCCTCCTCC   60
 M  A  A  A  S  S  I  S  I  G  S  T  V  P  R  A  S  S  S  S
TCCTCCTCCTCCTTACCGCAGTCAAGGGCACAAGCTGTCAACTTCAACTACTCCCTCCCT   120
 S  S  S  S  L  P  Q  S  R  A  Q  A  V  N  F  N  Y  S  L  P
CGTTTCACCGCTCTGAGGTCATCCACTCTCCTCTCTGGACCAGATTCCTCTTCTTTCGCC   180
 R  F  T  A  L  R  S  S  T  L  L  S  G  P  D  S  S  S  F  A
AAGTCTCTCCGCGGCTCCGTAACGAAACCTCAATCAACAGACACGAAGCCCTACGGATTG   240
 K  S  L  R  G  S  V  T  K  P  Q  S  T  D  T  K  P  Y  G  L
AACATCAACGCTTCGTACAAAGTGGCGGTCCTCGGTGCTGCCGGAGGGATCGGCCAGCCT   300
 N  I  N  A  S  Y  K  V  A  V  L  G  A  A  G  G  I  G  Q  P
CTGTCCCTTCTCATCAAAATGTCTCCTCTCGTCTCCACCCTCCACCTCTACGATATCGCC   360
 L  S  L  L  I  K  M  S  P  L  V  S  T  L  H  L  Y  D  I  A
AACGTCAAGGGAGTCGCCGCTGATCTGAGCCACTGCAACACTCCCTCTCAGGTCCGTGAT   420
 N  V  K  G  V  A  A  D  L  S  H  C  N  T  P  S  Q  V  R  D
TTCACCGGACCGGCTGAGCTGGCCGATTGTTTGAAAGATGTCAACGTCGTTGTCATCCCC   480
 F  T  G  P  A  E  L  A  D  C  L  K  D  V  N  V  V  V  I  P
GCTGGTGTGCCGAGAAAGCCCGGTATGACCCGTGACGATCTCTTCAACATCAACGCCGGT   540
 A  G  V  P  R  K  P  G  M  T  R  D  D  L  F  N  I  N  A  G
ATAGTGAAGACGCTTGTTGAGGCTGTCGCTGATAACTGTCCTAACGCCTTCATCCACATC   600
 I  V  K  T  L  V  E  A  V  A  D  N  C  P  N  A  F  I  H  I
ATCAGCAACCCTGTTAACTCCACCGTCCCCATTGCTGCTGAGGTGTTGAGGAAGAAAGGT   660
 I  S  N  P  V  N  S  T  V  P  I  A  A  E  V  L  R  K  K  G
GTCTATGATCCCAAGAAGCTCTTTGGCGTCACCACTTTGGATGTTGTGAGGGCGAACACC   720
 V  Y  D  P  K  K  L  F  G  V  T  T  L  D  V  V  R  A  N  T
TTTGTTTCTCAGAAAAAGAACTTGAAGCTCATCGATGTTGATGTTCCGGTCATCGGTGGC   780
 F  V  S  Q  K  K  N  L  K  L  I  D  V  D  V  P  V  I  G  G
CACGCTGGAATCACCATTCTGCCTCTTTTGTCGAAGACCAAGCCTTCGGTCAGCTTCACT   840
 H  A  G  I  T  I  L  P  L  L  S  K  T  K  P  S  V  S  F  T
GACGAAGAGATCGAGAAACTCACTGTGAGGATTCAGAACGCTGGAACCGAGGTGGTGGAT   900
 D  E  E  I  E  K  L  T  V  R  I  Q  N  A  G  T  E  V  V  D
GCTAAGGCGGGTGCTGGCTCGGCTACTTTGTCGATGGCGTATGCCGCAGCGAGATTTGTG   960
 A  K  A  G  A  G  S  A  T  L  S  M  A  Y  A  A  A  R  F  V
GAGTCGTCTCTTCGTGCTCTTGACGGAGATGGAGATGTTTACGAGTGCTCTTTCGTGGAC   1020
 E  S  S  L  R  A  L  D  G  D  G  D  V  Y  E  C  S  F  V  D
TCTACTCTCACTGATCTTCCTTTCTTTGCATCACGGATCAAGATTGGGAGGAACGGAGTT   1080
 S  T  L  T  D  L  P  F  F  A  S  R  I  K  I  G  R  N  G  V
GAAGCTGTGATTGAGTCTGACCTCCAAGGGCTGACTGAGTATGAGCACAAGGCGTTAGAA   1140
 E  A  V  I  E  S  D  L  Q  G  L  T  E  Y  E  H  K  A  L  E
GCTCTTAAGCCTGAACTGAAAGCTAGCATCGAAAAGGGTGTTGCATTCGCAAACAAACCT   1200
 A  L  K  P  E  L  K  A  S  I  E  K  G  V  A  F  A  N  K  P
GCTAACTAA                                                      1209
 A  N
```

Supplementary Figure 1. The full length cDNA and predicted amino acid sequence of *BraMDH*.

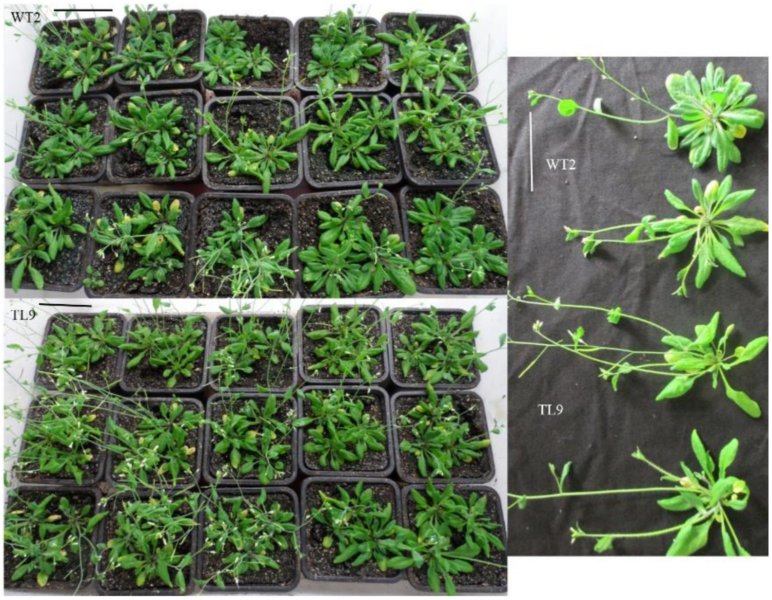

Supplementary Figure 2. Bolting in wild-type (WT2) and homozygous transgenic (TL9) *Arabidopsis* plants. Bolting of WT2 and TL9 plants after 35 days of growth in a substrate mixture of peat, vermiculite, and pearlite (3:1:0.5, v/v). Bar = 5 cm.

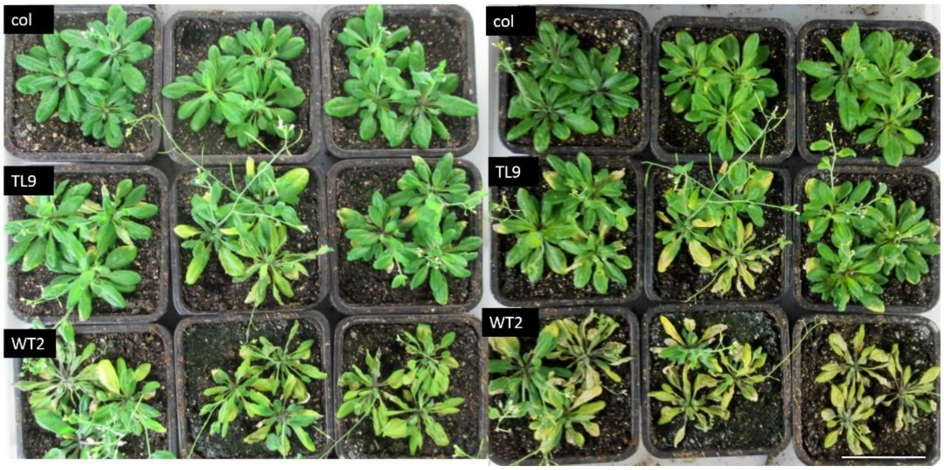

Supplementary Figure 3. Effect of aluminum stress on the rosette-stage wild-type (WT2) and homozygous transgenic (TL9) *Arabidopsis* plants. Rosette-stage WT2 and TL9 plants treated with 150 μM $AlCl_3$ (Treatment 2) for 2 days (left) and 5 days (right). Col, untreated wild-type plants; WT, treated wild-type plants; TL, treated transgenic plants. Bar = 5 cm.

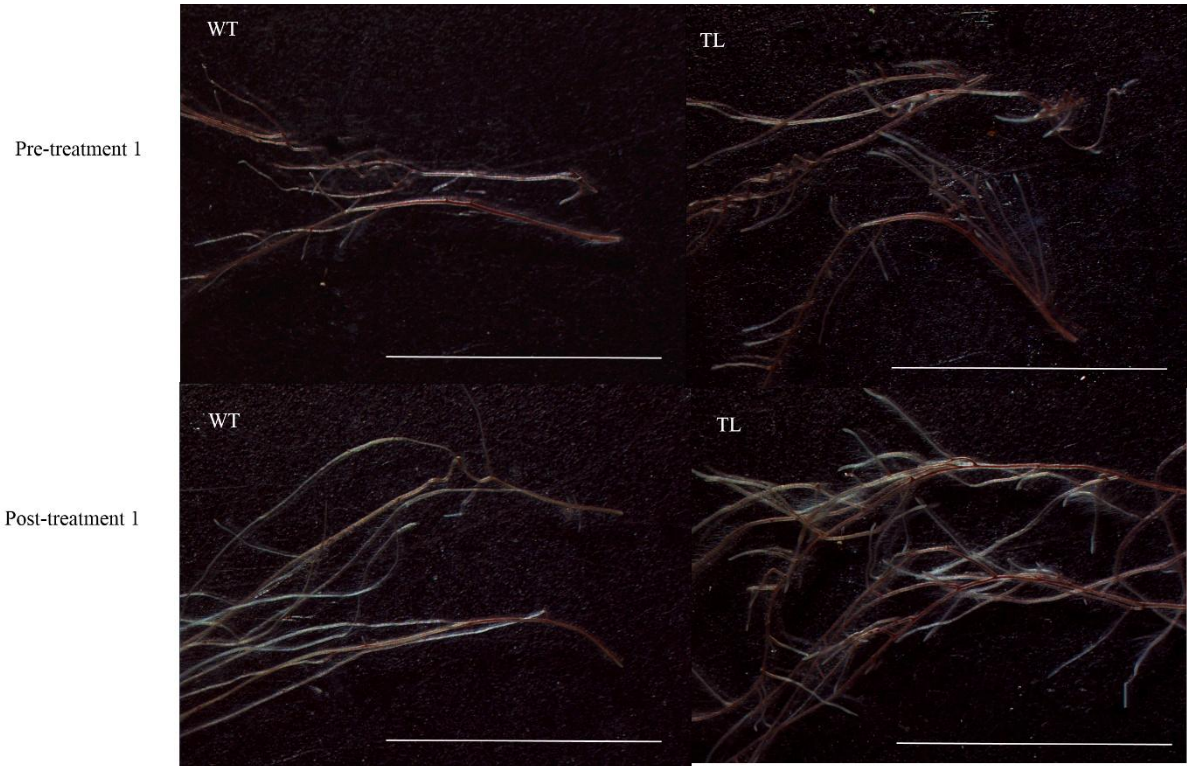

Supplementary Figure 4. The root activity determination of pre- and post-treatment wild-type (WT) and homozygous transgenic (TL) seedlings using triphenyl tetrazolium chloride reduction method. The root hairs of WT and TL seedlings before Treatment 1, and that of WT and TL seedlings after 3-day Treatment 1. Bar = 1 cm.
